# Dry cow pasture silage mineral concentrations and dry cow mineral requirements and supplementation practices on Irish dairy farms

**DOI:** 10.1186/s13620-025-00310-6

**Published:** 2025-10-15

**Authors:** Louise Horan, Rachel Reardon, Ángel García-Muñoz, John F. Mee, Joseph Patton, Michael Dineen, Emily M. Sitko, Ainhoa Valldecabres

**Affiliations:** 1 Teagasc, Animal & Grassland Research and Innovation Centre, Fermoy, Co. Cork, Moorepark, Ireland; 2 School of Veterinary Medicine, University College Dublin, Belfield, Dublin 4, Dublin, D04 W6F6 Ireland; 3 Facultad de Veterinaria, Universidad Cardenal Herrera-CEU, CEU Universities, Valencia, Spain

**Keywords:** Dry period, Mineral nutrition, Grazing cow, Milk fever

## Abstract

In the Republic of Ireland, pregnant dry dairy cows are typically housed and fed pasture silage. However, its mineral concentrations vary greatly, and dietary mineral recommendations are based on other main feedstuffs and cow types. This study, which is part of a larger project, aims to describe pasture silage mineral composition, comparing it to estimated Irish dry cow requirements, and describe mineral feeding practices. Macrominerals and trace minerals were determined for pasture silage samples from 27 commercial farms. Farmers completed a feeding management questionnaire, and mineral requirements for each farm were calculated for pregnant dry cows at 270 d of gestation, with mean estimated live weight of 557 kg and dry matter (DM) intake of 9.2 kg/d. Mineral concentrations in pasture silage exceeded the estimated dietary requirements for all macrominerals and some trace minerals (samples exceeding; Ca, Cl, K, Mg, Na, P, S, and Fe: 100.0%, Mn: 88.9%, and Co: 55.6%), while it didn’t meet them for some trace minerals (samples not meeting; Cu, I and Se: 100.0%, and Zn: 51.9%). Calculated pasture silage dietary cation-anion difference ranged from 18 to 49 mEq/100 g DM. All farms supplemented minerals; 91.6% provided a multi-mineral product (types/brands varied), which was mostly top-dressed over pasture silage (77.8% of farms) once a day (51.9% of farms). Only 26.0% of farms tested pasture silage for mineral composition, the importance of which should be promoted. Our estimated requirements and findings should be considered along with recommendations for disease prevention for dry cow mineral feeding optimization.

**Supplementary Information:**

The online version contains supplementary material available at 10.1186/s13620-025-00310-6.

## Introduction

Dairy farming in the Republic of Ireland is predominately a spring-calving pasture-based production system, typically involving cows grazing outdoors for most of the year and being housed during the winter months which coincides with the non-lactating, “dry” period. Pasture silage is the main forage provided to dry cows during these housed months, however, mineral composition of this forage tends to vary greatly [1, 2]. Mineral nutrition during the dry period plays a key role in postpartum performance, including metabolic and infectious disease incidence. In a recent Irish survey, farmers reported that milk fever, which may result from inadequate (deficit or excess) intake of macrominerals that can interfere with Ca metabolism (i.e. Ca, Mg, K or P [3–6]) was a “significant (regularly treating severe cases with some cows lost/culled) or routine (regularly treating cows to control issues) problem” in their herds (15.7%; 82/522 [7]). Some trace minerals such as Se (frequently low in forages [8]), Cu, and Zn are key to immune system function (determinant for infectious disease resolution [9]) and reproduction performance [10], which is particularly important in seasonal calving systems where a prompt return to cyclicity and a high pregnancy rate are necessary to achieve a compact calving pattern. Hence, determining the mineral composition of pasture silage is critical to formulating a mineral supplementation plan which accounts for any mineral deficiencies, excesses, or antagonists with potential to impair cows’ performance. Optimizing mineral supplementation protocols is critical to ensure adequate mineral nutrition of dry cows. Current dietary mineral recommendations for dry cows apply to cows consuming either fresh pasture (New Zealand recommendations [11]) or total mixed rations (National Academies of Science, Engineering, and Medicine (NASEM) recommendations from the US [12]) and dairy cows which may be genotypically and phenotypically divergent from Irish cows. Despite the importance of dry cow mineral nutrition, Republic of Ireland specific data is limited to a database, excluding Cl and dietary cation-anion difference (DCAD), describing mineral concentrations in Irish pasture silage samples collected and tested between 1990 and 1993 [2]. Additionally, only a survey including two questions on dry cow mineral provision (length of time cows receive minerals and type of minerals) addressed dry cow mineral feeding practices in Irish dairy farms [13]. Thus, the objectives of this study were to (1) describe pasture silage mineral composition and compare it to estimated dry cow mineral requirements, and (2) describe mineral feeding practices in commercial spring-calving, pasture-based Irish dairy farms.

## Materials and methods

This study was part of a larger project investigating transition cow health and management. In brief, study herds were chosen from the respondents to a transition cow health and management survey distributed among Teagasc Dairy Advisory clients which tend to operate at a higher standard of technical performance than the overall dairy farmer population [7, 14]. There were 601 respondents but only those who met the following eligibility criteria for participation in the on-farm study were included in the stratified random sampling process that was used to select the study participants (*n* = 119): (1) Spring-calving, (2) Milking herd size >60 cows, (3) Located within a 2 h drive from Teagasc Moorepark, (4) Irish Cattle Breeding Federation (ICBF) client with an active HerdPlus subscription, (5) Minimum of 4 milk recordings completed in 2022, and (6) Provided consent for Teagasc to access their ICBF profile and for the farmer to be contacted about the on-farm study. Strata were defined for milk yield quartiles and bought-in concentrate feed per cow (based on pre-defined categories provided in the survey; ≤ or >1 tonne). Based on power calculations for meeting the objectives of the larger project and not this study, 27 farms were randomly selected from the strata.

A representative sample of pasture silage provided to dry cows on each farm was collected using the star uni-forage sampler with crank handle and spiral assist (Star Quality Samplers, Edmonton, AB, Canada) during one farm visit performed in January - February 2023. This was done by taking 9 samples in a W shaped pattern across the open face of the pasture silage pit and, where farmers were feeding pasture silage bales, by taking a sample from each bale being opened on the day of the visit. Samples from the same farm were combined and placed into a sealed, labelled plastic bag manually devoid of air. Samples were delivered fresh, within 24 h after collection, to FBA Laboratories (Cappoquin, Co. Waterford, Ireland) for mineral determination. Samples were dried in an oven at 60˚C for 72 h. Dried samples were ground at 1 mm and mineral concentrations (Ca, Co, Cu, I, Fe, Mg, Mn, Mo, P, K, Se, Na, S, and Zn) were determined by inductively coupled mass spectrometry (NexION 2000 ICP-MS; PerkinElmer Inc., Shelton, CT, USA and Agilent 7700 ICP-MS; Agilent Technologies Inc., Santa Clara, CA, USA) except for Cl which was determined using a titration method [15]. Additionally, neutral detergent fibre (NDF) determination, required for the dry matter (DM) intake estimation used in the calculation of mineral requirements described below, was performed using Filter Bag Technology (Ankom 220 Fiber Analyzer; Ankom Technology Corp., Macedon, NY, USA). The DCAD was calculated as: (Na + K) − (Cl + S) in mEq/100 g DM.

A dry cow nutrition and management questionnaire addressing how farmers’ implemented dry cow management practices relating to nutrition, housing, drying off, and health was completed in an oral interview with participating farmers; only pasture silage testing and mineral feeding questions (*n* = 3) are reported and discussed here. The questions were: (1) What method of distribution is used for providing the dry cow mineral to stock?, (2) How often is the dry cow mineral distributed to stock?, and (3) Has silage that is fed to dry cows been tested?.

The questionnaire was designed using SurveyMonkey (SurveyMonkey Inc. San Mateo, CA, USA). Responses were manually recorded and summarized using Microsoft Excel (Microsoft Corp., Redmond, WA, USA). Similar responses were combined where appropriate. Details (product labels) of dry cow mineral supplements were collected during the visit in 24 of the 27 study herds. Descriptive data analysis was performed using Excel.

Mineral requirements for mature pregnant dry cows (at 270 d of gestation) were estimated for each farm using the estimated live weight (determined using each herd’s maintenance sub index from ICBF [16] and the Maintenance Excel Calculator [17]), the estimated daily DM intake (determined from the estimated live weights, pasture silage NDF values, and Eqs. 12 − 1 from NASEM 2021 [12]) and the respective equations and absorption coefficients available for each of the minerals from NASEM 2021 [12]. The mean estimated dietary mineral requirement of the 27 farms is used throughout this report.

## Results and discussion

Mean [± standard error of the mean (SEM)] herd size was 151 ± 93 cows (range = 61 to 383 cows), 305-d milk yield was 6,683 ± 890 kg (range = 3,782 to 11,417 kg), and calving interval was 369 ± 7 d (range = 360 to 378 d). Fifteen farmers reported to buy ≤ 1 tonne and twelve farmers reported to buy >1 tonne of concentrate feed/cow annually. Mean herd size and 305-d milk yield for farms in this study were 62.4% and 16.9% higher than the national mean (93 cows and 5,716 kg/cow [17]). Most farms enrolled in this study were located in the south of the Republic of Ireland, the region with the highest number of dairy cows and with soil characteristics different to other regions [18, 19], potentially influencing pasture silage composition and dry cow management [20].

Pasture silage mineral composition is presented in Table 1. Results show a large variation across samples and agree with previous Irish research [1, 2]. The most variable mineral was Fe [coefficient of variation (CV) = 64.0%] while the least variable mineral was S (CV = 16.9%; Table 1). Grass silage Fe concentration is mainly increased due to soil contamination, suggesting that the wide observed concentration variation is best explained by differences in soil contamination risk at harvest [21]. Table 2 shows the mean and range of estimated mineral requirements for mature pregnant dry cows at 270 d of gestation with a mean estimated live weight of 557 kg and a mean estimated DM intake of 9.2 kg/d, while estimated requirements by farm are presented in the Supplementary Tables S1. Results show similar mineral requirements for each farm, however, the variability in mineral concentrations of silage samples between farms indicates that requirements are often exceeded or not met by pasture silage alone. The sampled pasture silage exceeded estimated dietary requirements for all macrominerals and some trace minerals (proportion of samples exceeding; Ca, Cl, K, Mg, Na, P, S, and Fe: 100.0%, Mn: 88.9%, and Co: 55.6%) and didn’t meet them for some trace minerals (proportion of samples not meeting; Cu, I and Se: 100.0%, and Zn: 51.9%). It should be noted that dietary mineral antagonists were not accounted for in the mineral requirement calculations, except for Mg, where K concentration was accounted for, hence availability of minerals for absorption could have been overestimated and the true dietary requirements underestimated.

**Table 1:** Mineral concentrations on a DM basis of pasture silage fed to pregnant dry cows on 27 Irish dairy farms. The number of decimal places reported reflects the precision of the laboratorial meathods used

Mineral	Mean	Median	Range			SD^1^	CV^1^
Macrominerals, %							
Ca	0.61	0.60	0.40	-	0.99	0.14	22.8
Cl	0.86	0.90	0.50	-	1.30	0.19	21.7
K	1.99	1.92	0.88	-	2.99	0.50	25.5
Mg	0.17	0.16	0.11	-	0.23	0.03	27.3
Na	0.39	0.39	0.17	-	0.81	0.14	34.0
* P*	0.28	0.29	0.20	-	0.40	0.05	17.2
S	0.21	0.21	0.15	-	0.30	0.04	16.9
Trace minerals, mg/kg							
Cu	7.3	7.4	5.1	-	11.5	1.5	20.9
Fe	435	389	106	-	1351	279	64.0
Mn	106	93	27	-	329	60	57.0
Zn	31	30	21	-	45	6	18.9
Co	0.16	0.16	0.06	-	0.38	0.08	46.7
I	0.25	0.22	0.11	-	0.50	0.10	41.4
Se	0.07	0.07	0.02	-	0.18	0.03	48.5
Mo	1.4	1.1	0.5	-	2.9	0.7	50.1
DCAD^1^, mEq/100 g	31	29	18	-	49	8	25.4

^1^*SD*, Standard deviation, *CV* Coefficient of variation (%), *DCAD* Dietary cation-anion difference calculated as: (Na + K) − (Cl + S)

All farmers provided mineral supplements, each using 1 (63.0%), 2 (34.6%) or 3 different products (3.8%). Overall, farmers used a multi-mineral product (types/brands varied; 91.6%), sometimes complemented with an additional Mg source (all MgCl_2_ flakes; 16.7%). Mineral composition of the supplements provided on 24 of the study farms is summarized in supplementary Table S2. Most farmers top-dressed the dry cow minerals (on top of pasture silage; 77.8%), three mixed the minerals with forage using a diet feeder (11.1%), two fed minerals through the drinking water (7.4%), and one only provided the minerals as a bucket lick (3.7%). Frequency of dry cow mineral distribution varied by form of mineral used and farm; top-dressed minerals were provided once a day (51.9%), twice a day (33.3%), or when feed was delivered (3.7%; every 3 days for one specific farm); minerals were continuously available through the water troughs (7.4%) and bucket licks (3.7%; replenished according to consumption). However, only 26.0% of farmers reported testing pasture silage designated for their dry cows for mineral concentrations, suggesting that mineral supplementation plans were not established according to the main feedstuff’s mineral composition. The remaining farmers were either not testing their pasture silage (37.0%) or testing pasture silage for nutritive value only (37.0%).

**Table 2:** Mean estimated dietary mineral concentration requirements on a dry matter (DM) basis for mature pregnant dry cows at 270 d of gestation on 27 Irish dairy farms^1^

**Mineral**	**Mean dietary concentration required**^1^
Macrominerals, %	
Ca	0.42
Cl	0.13
K	0.68
Mg	0.13
Na	0.16
P	0.23
S	0.20
Trace minerals, mg/kg	
Cu	20.26
Fe	15.07
Mn	45.52
Zn	30.12
Co	0.20
I	0.66
Se	0.30

Mature pregnant dry cows on the 27 farms had a mean estimated live weight of 557 kg and daily pasture silage DM intake of 9.2 kg. Refer to supplementary Tables S1 and S2 for a full breakdown of estimated DM intake, live weight, and mineral requirements by farm

It should be noted that estimated mineral requirements differ from recommendations for disease prevention. For instance, estimated Mg requirements (0.13% DM) would be met by pasture silage alone, however, its recommended concentration for transition cow health optimization and milk fever prevention is 0.4% DM [22]. Unsurprisingly, all mineral supplement regimes evaluated in this study provided Mg, however, the actual dry cow intake of Mg cannot be calculated from this study. A quadratic association between prepartum dietary Ca concentration and milk fever incidence has been described [6], so that low (< 0.8% DM) and high concentrations (>1.5% DM) may result in lower milk fever incidence but intermediate concentrations (1.2% DM) may result in high milk fever incidence ([22], and as reviewed by [23]). Pasture silage Ca concentration (range = 0.40 to 0.99% DM) exceeded estimated dietary Ca requirements (0.42% DM) in all samples, and 62.5% of study farms also provided Ca in the mineral supplement (Supplementary Table S2) potentially increasing the total amount of absorbed Ca. Pasture silage P content (range = 0.20 to 0.40% DM) exceeded the estimated dietary requirement (0.23% DM) in all samples, while the mean concentration was equal to the published recommendation of ≤ 0.3% DM for the reduction of hypocalcaemia [24].

Mean K concentration of pasture silage samples (1.99%) was similar to the recommendation (< 2.0% DM) for the reduction of milk fever risk [25], while the highest concentration observed was 2.99% DM, suggesting that adverse effects of high K (reduction of Mg absorption and contribution to high DCAD) could be mitigated by the addition of anionic salts or dilution with a lower K forage (e.g. wheat straw or maize silage [12]), provided that other minerals essential to Ca metabolism are within required concentrations. Calculated pasture silage DCAD according to the standard formula considering Na, K, Cl, and S was notably higher (range = 18 to 49 mEq/100 g DM) than values recommended for hypocalcaemia prevention (−10 to −15 mEq/100 g DM [26]), and anions were not provided in the mineral supplements to decrease this value (Supplementary Table S2). It should be noted that accounting for Mg in the DCAD calculation may further increase its value.

While the study has provided valuable insights on silage mineral concentrations and dry cow mineral nutrition in spring-calving pasture-based dairy farms in the Republic of Ireland, it is important to acknowledge the limitations inherent in its design. First, the study sample size was not estimated to meet the objectives of this study. And second, study farms were clients of Teagasc and located in the south of the Republic of Ireland. Choosing survey participants from members of an agricultural organisation, like Teagasc, and from a specific geographic location can omit certain farmer and farm demographics likely to affect the outcome [27], limiting the widespread applicability of the study findings.

In conclusion, there was wide variation in pasture silage mineral composition and implemented mineral feeding practices across the 27 study farms. As such, dry cow feed mineral concentrations (pasture silage and supplements) may be contributing to metabolic disorders on individual farms across the Republic of Ireland. With, only 26.0% of farmers testing their pasture silage for mineral concentrations there is opportunity to improve metabolic outcomes on farm. The importance of feed testing should be promoted to farmers through advisory and/or dissemination activities to allow for the optimisation of dry cow mineral nutrition.

## Supplementary Information

Supplementary Material 1: Supplementary material: Dry cow pasture silage mineral concentrations and dry cow mineral requirements and supplementation practices on Irish dairy farms. Description of data: Two tables which include 1) the dry cow mineral concentration requirements by farm and 2) the composition of mineral supplements used by farm.

## Data Availability

The datasets used and analysed during the current study are available from the corresponding author on reasonable request.

## Abbreviations

CV: Coefficient of Variation
DCAD: Dietary Cation-Anion Difference
DM: Dry Matter
ICBF: Irish Cattle Breeding Federation
NASEM: National Academies of Science, Engineering, and Medicine
NDF: Neutral Detergent Fibre
SD: Standard Deviation
SEM: Standard Error of the Mean

## References

[1] Patterson JD, Sahle B, Gordon AW, Archer JE, Yan T, Grant N, et al. Grass silage composition and nutritive value on Northern Ireland farms between 1998 and 2017. Grass Forage Sci. 2021;76:300–8. 10.1111/gfs.12534.

[2] Rogers P, Murphy W. Levels of dry matter, major elements (calcium, magnesium, nitrogen, phosphorus, potassium, sodium and sulphur) and trace elements (cobalt, copper, iodine, manganese, molybdenum, selenium and zinc) in Irish grass, silage and hay. 2000 [cited 27 Feb 2024]. Available: http://homepage.tinet.ie/~progers/0forage.htm

[3] Van de Braak AE, Van ’T, Klooster AT, Malestein A. Influence of a deficient supply of magnesium during the dry period on the rate of calcium mobilisation by dairy cows at parturition. Res Vet Sci. 1987;42:101–8. 3103179

[4] Fisher LJ, Dinn N, Tait RM, Shelford JA. Effect of level of dietary potassium on the absorption and excretion of calcium and magnesium by lactating cows. Can J Anim Sci. 1994;74:503–9. 10.4141/cjas94-071.

[5] Cohrs I, Wilkens MR, Grünberg W. Short communication: effect of dietary phosphorus deprivation in late gestation and early lactation on the calcium homeostasis of periparturient dairy cows. J Dairy Sci. 2018;101:9591–8. 10.3168/jds.2018-14642. 30100496 10.3168/jds.2018-14642

[6] Oetzel GR. Meta-Analysis of nutritional risk factors for milk fever in dairy cattle. J Dairy Sci. 1991;74:3900–12. 10.3168/jds.S0022-0302(91)78583-4. 1661751 10.3168/jds.S0022-0302(91)78583-4

[7] Horan L, Patton J, McAloon CG, García-Muñoz Á, Regan Á, Mee JF et al. Transition cow health and management in pasture-based dairy herds: a farmers’ survey. PLoS One. 2024;19:e0314987. 10.1371/journal.pone.0314987. 10.1371/journal.pone.0314987 PMC11651598 39689143

[8] Culleton N, Parle PJ, Rogers PAM, Murphy WE, Murphy J. Selenium supplementation for dairy cows. Ir J Agric Food Res. 1997;36:23–9.

[9] Ingvartsen KL, Moyes K. Nutrition, immune function and health of dairy cattle. Animal. 2013;7:112–22. 10.1017/S175173111200170X. 23031687 10.1017/S175173111200170X

[10] Corbett RB. Trace mineral nutrition in confinement dairy cattle. Vet Clin North Am - Food Anim Pract. 2023;39:425–38. 10.1016/j.cvfa.2023.06.004. 37587002 10.1016/j.cvfa.2023.06.004

[11] Holmes CW, Brookes IM, Garrick DJ, Mackenzie DDS, Parkinson T, Wilson G. Milk production from pasture principles and practices. 2nd ed. Massey University. 2002.

[12] NRC. Nutrient requirements of dairy cattle: eighth revised edition. National Academies of Sciences, Engineering, and Medicine, Division on Earth and Life Studies; Board on Agriculture and Natural Resources; Committee on Nutrient Requirements of Dairy Cattle. 2021. 38386771

[13] Cummins C, Berry DP, Sayers R, Lorenz I, Kennedy E. Questionnaire identifying management practices surrounding calving on spring-calving dairy farms and their associations with herd size and herd expansion. Anim. 2016;10:868–77. 10.1017/S1751731116000124 10.1017/S1751731116000124 26857400

[14] Bogue P. Impact of participation in Teagasc dairy discussion groups. 2013 [cited 1 Mar 2024]. Available: https://www.teagasc.ie/media/website/publications/2013/Discussion_Group_Report_Web_Jan2013.pdf

[15] Gilliam JW. Rapid measurement of Chlorine in plant materials. Soil Sci Soc Am J. 1971;35:512–3. 10.2136/sssaj1971.03615995003500030051x.

[16] Irish Cattle Breeding Federation. 2024.

[17] Dillon E, Donnellan T, Moran B, Lennon J. Teagasc national farm survey 2022. 2023 [cited 14 Jun 2025]. Available: https://teagasc.ie/wp-content/uploads/media/website/publications/2023/NFSfinalreport2022.pdf

[18] CSO. Census of Agriculture 2020 - Preliminary Results; Livestock. 2020.

[19] Creamer R, O’Sullivan L. The soils of Ireland. Springer Int Publishing AG. 2018. 10.1007/978-3-319-71189-8_13.

[20] Fox I. Soil properties, lime and phosphorus fertiliser management affecting grass production in Ireland. Queen’s University Belfast; 2020.

[21] Hansen SL, Spears JW. Bioaccessibility of iron from soil is increased by silage fermentation. J Dairy Sci. 2009;92:2896–905. 10.3168/jds.2008-1933. 19448021 10.3168/jds.2008-1933

[22] Goff JP. Macromineral disorders of the transition cow. Vet Clin Food Anim Pract. 2004;20:471–94. 10.1016/j.cvfa.2004.06.003. 10.1016/j.cvfa.2004.06.003 15471621

[23] Martín-Tereso J, Verstegen MWA. A novel model to explain dietary factors affecting hypocalcaemia in dairy cattle. Nutr Res Rev. 2011;24:228–43. 10.1017/S0954422411000126 22098692 10.1017/S0954422411000126

[24] Lean IJ, DeGaris PJ, McNeil DM, Block E. Hypocalcemia in dairy cows: Meta-analysis and dietary cation anion difference theory revisited. J Dairy Sci. 2006;89:669–84. 10.3168/jds.S0022-0302(06)72130-0. 16428636 10.3168/jds.S0022-0302(06)72130-0

[25] Goff JP, Horst RL. Factors to concentrate on to prevent periparturient disease in the dairy cow with special emphasis on milk fever. American Association of Bovine Practitioners Conference Proceedings. 1998. pp. 154–63. 10.21423/aabppro19985698.

[26] Santos JEP, Lean IJ, Golder H, Block E. Meta-analysis of the effects of prepartum dietary cation-anion difference on performance and health of dairy cows. J Dairy Sci. 2019;102:2134–54. 10.3168/jds.2018-14628. 30612801 10.3168/jds.2018-14628

[27] Emerson H, MacFarlane R, COMPARATIVE BIAS BETWEEN SAMPLING FRAMES FOR FARM SURVEYS. J Agric Econ. 1995;46:241–51. 10.1111/j.1477-9552.1995.tb00770.x.

