# Supplementary material for: Dry cow pasture silage mineral concentrations and dry cow mineral requirements and supplementation practices on Irish dairy farms
Source: Ir Vet J. 2025 Oct 15;78:23. doi: 10.1186/s13620-025-00310-6 (PMC12523131; doi:10.1186/s13620-025-00310-6)
Supplement: Supplementary file 1 [file 13620_2025_310_MOESM1_ESM.docx]

Supplementary material: Dry cow pasture silage mineral concentrations and dry cow mineral requirements and supplementation practices on Irish dairy farms

Rachel Reardon1†, Louise Horan1,2†, Ángel García-Muñoz3, John F. Mee1, Joseph Patton1, Michael Dineen1, Emily M. Sitko1 & Ainhoa Valldecabres1,3*

1Teagasc, Animal & Grassland Research and Innovation Centre, Moorepark, Fermoy, Co. Cork, Ireland.

2School of Veterinary Medicine, University College Dublin, Belfield, Dublin 4, Dublin D04 W6F6, Ireland

3Facultad de Veterinaria, Universidad Cardenal Herrera-CEU, CEU Universities, Valencia, Spain.

*Corresponding author: ainhoa.valldecabres@uchceu.es

† Rachel Reardon and Louise Horan are joint first authors, both authors contributed equally to this work.

Table S1. Estimated dry matter (DM) intake, live weight (LW), and dietary macromineral concentration requirements on a DM basis for mature pregnant dry cows at 270 d of gestation on 27 Irish dairy farms

|  | Dietary requirement, total (% of DM)1 |
| --- | --- |
| Farm | DMI1 | LW2 | Ca | Cl | K | Mg | Na | P | S |
| 1 | 9.7 | 586 | 0.43 | 0.13 | 0.68 | 0.12 | 0.16 | 0.23 | 0.20 |
| 2 | 9.4 | 561 | 0.42 | 0.13 | 0.68 | 0.14 | 0.16 | 0.23 | 0.20 |
| 3 | 7.8 | 471 | 0.43 | 0.13 | 0.68 | 0.14 | 0.16 | 0.23 | 0.20 |
| 4 | 9.7 | 591 | 0.43 | 0.13 | 0.68 | 0.13 | 0.16 | 0.23 | 0.20 |
| 5 | 9.1 | 551 | 0.43 | 0.13 | 0.68 | 0.13 | 0.16 | 0.23 | 0.20 |
| 6 | 7.9 | 481 | 0.43 | 0.13 | 0.68 | 0.13 | 0.16 | 0.23 | 0.20 |
| 7 | 8.2 | 491 | 0.42 | 0.13 | 0.68 | 0.13 | 0.16 | 0.23 | 0.20 |
| 8 | 10.0 | 601 | 0.42 | 0.13 | 0.68 | 0.13 | 0.16 | 0.23 | 0.20 |
| 9 | 9.4 | 571 | 0.43 | 0.13 | 0.68 | 0.14 | 0.16 | 0.23 | 0.20 |
| 10 | 9.7 | 586 | 0.43 | 0.13 | 0.68 | 0.14 | 0.16 | 0.23 | 0.20 |
| 11 | 9.3 | 556 | 0.42 | 0.13 | 0.68 | 0.14 | 0.16 | 0.23 | 0.20 |
| 12 | 9.5 | 566 | 0.42 | 0.13 | 0.68 | 0.14 | 0.16 | 0.23 | 0.20 |
| 13 | 9.7 | 586 | 0.43 | 0.13 | 0.68 | 0.12 | 0.16 | 0.23 | 0.20 |
| 14 | 9.5 | 571 | 0.42 | 0.13 | 0.68 | 0.13 | 0.16 | 0.23 | 0.20 |
| 15 | 9.9 | 601 | 0.43 | 0.13 | 0.68 | 0.12 | 0.16 | 0.23 | 0.20 |
| 16 | 8.2 | 496 | 0.43 | 0.13 | 0.68 | 0.13 | 0.16 | 0.23 | 0.20 |
| 17 | 9.8 | 596 | 0.43 | 0.13 | 0.68 | 0.13 | 0.16 | 0.23 | 0.20 |
| 18 | 9.2 | 556 | 0.43 | 0.13 | 0.68 | 0.13 | 0.16 | 0.23 | 0.20 |
| 19 | 9.7 | 586 | 0.43 | 0.13 | 0.68 | 0.14 | 0.16 | 0.23 | 0.20 |
| 20 | 9.7 | 586 | 0.43 | 0.13 | 0.68 | 0.11 | 0.16 | 0.23 | 0.20 |
| 21 | 9.5 | 571 | 0.42 | 0.13 | 0.68 | 0.14 | 0.16 | 0.23 | 0.20 |
| 22 | 9.1 | 551 | 0.43 | 0.13 | 0.68 | 0.13 | 0.16 | 0.23 | 0.20 |
| 23 | 9.9 | 591 | 0.42 | 0.13 | 0.68 | 0.13 | 0.16 | 0.23 | 0.20 |
| 24 | 9.7 | 586 | 0.42 | 0.13 | 0.68 | 0.14 | 0.16 | 0.23 | 0.20 |
| 25 | 9.3 | 561 | 0.42 | 0.13 | 0.68 | 0.13 | 0.16 | 0.23 | 0.20 |
| 26 | 8.4 | 506 | 0.42 | 0.13 | 0.68 | 0.13 | 0.16 | 0.23 | 0.20 |
| 27 | 8.2 | 486 | 0.42 | 0.13 | 0.67 | 0.14 | 0.16 | 0.23 | 0.20 |

1Dry matter intake was estimated from the predicted live weight and grass silage NDF concentration using equation 12-1 from NASEM 2021 in kg/d. Mineral requirements were estimated using the respective equations and absorption coefficients from NASEM 2021 [1].

2 Live weight was estimated using each herd maintenance sub index from ICBF [2] and a maintenance excel calculator [3] in kg.

Table S2. Estimated dry matter (DM) intake, live weight (LW), and dietary trace mineral requirements on a DM basis for mature pregnant dry cows at 270 d of gestation on 27 Irish dairy farms

|  | Dietary requirement, total (mg/kg of DM)1 |
| --- | --- |
| Farm | DMI1 | LW2 | Cu | Fe | Mn | Zn | Co | I | Se |
| 1 | 9.7 | 586 | 20.40 | 15.18 | 45.85 | 30.16 | 0.20 | 0.65 | 0.30 |
| 2 | 9.4 | 561 | 19.99 | 14.87 | 44.92 | 30.06 | 0.20 | 0.65 | 0.30 |
| 3 | 7.8 | 471 | 20.42 | 15.19 | 45.88 | 30.17 | 0.20 | 0.72 | 0.30 |
| 4 | 9.7 | 591 | 20.39 | 15.17 | 45.81 | 30.16 | 0.20 | 0.64 | 0.30 |
| 5 | 9.1 | 551 | 20.41 | 15.19 | 45.87 | 30.16 | 0.20 | 0.67 | 0.30 |
| 6 | 7.9 | 481 | 20.35 | 15.14 | 45.74 | 30.15 | 0.20 | 0.71 | 0.30 |
| 7 | 8.2 | 491 | 20.24 | 15.06 | 45.49 | 30.12 | 0.20 | 0.70 | 0.30 |
| 8 | 10.0 | 601 | 20.23 | 15.06 | 45.47 | 30.12 | 0.20 | 0.63 | 0.30 |
| 9 | 9.4 | 571 | 20.41 | 15.19 | 45.86 | 30.16 | 0.20 | 0.66 | 0.30 |
| 10 | 9.7 | 586 | 20.32 | 15.12 | 45.66 | 30.14 | 0.20 | 0.65 | 0.30 |
| 11 | 9.3 | 556 | 20.20 | 15.03 | 45.38 | 30.11 | 0.20 | 0.66 | 0.30 |
| 12 | 9.5 | 566 | 20.12 | 14.97 | 45.22 | 30.09 | 0.20 | 0.65 | 0.30 |
| 13 | 9.7 | 586 | 20.34 | 15.13 | 45.71 | 30.15 | 0.20 | 0.65 | 0.30 |
| 14 | 9.5 | 571 | 20.30 | 15.11 | 45.62 | 30.14 | 0.20 | 0.65 | 0.30 |
| 15 | 9.9 | 601 | 20.42 | 15.19 | 45.88 | 30.17 | 0.20 | 0.64 | 0.30 |
| 16 | 8.2 | 496 | 20.42 | 15.20 | 45.89 | 30.17 | 0.20 | 0.70 | 0.30 |
| 17 | 9.8 | 596 | 20.41 | 15.19 | 45.87 | 30.16 | 0.20 | 0.64 | 0.30 |
| 18 | 9.2 | 556 | 20.42 | 15.19 | 45.88 | 30.17 | 0.20 | 0.66 | 0.30 |
| 19 | 9.7 | 586 | 20.40 | 15.18 | 45.85 | 30.16 | 0.20 | 0.65 | 0.30 |
| 20 | 9.7 | 586 | 20.34 | 15.13 | 45.71 | 30.15 | 0.20 | 0.65 | 0.30 |
| 21 | 9.5 | 571 | 20.20 | 15.03 | 45.38 | 30.11 | 0.20 | 0.65 | 0.30 |
| 22 | 9.1 | 551 | 20.41 | 15.19 | 45.87 | 30.16 | 0.20 | 0.67 | 0.30 |
| 23 | 9.9 | 591 | 20.10 | 14.95 | 45.16 | 30.08 | 0.20 | 0.64 | 0.30 |
| 24 | 9.7 | 586 | 20.24 | 15.06 | 45.47 | 30.12 | 0.20 | 0.64 | 0.30 |
| 25 | 9.3 | 561 | 20.29 | 15.10 | 45.59 | 30.13 | 0.20 | 0.66 | 0.30 |
| 26 | 8.4 | 506 | 20.24 | 15.06 | 45.48 | 30.12 | 0.20 | 0.69 | 0.30 |
| 27 | 8.2 | 486 | 19.96 | 14.85 | 44.86 | 30.05 | 0.20 | 0.69 | 0.30 |

1Dry matter intake was estimated from the predicted live weight and grass silage NDF concentration using equation 12-1 from NASEM 2021 in kg/d. Mineral requirements were estimated using the respective equations and absorption coefficients from NASEM 2021 [1].

2 Live weight was estimated using each herd maintenance sub index from ICBF [2] and a maintenance excel calculator [3] in kg.

Table S3. Summary statistics for the composition of dry cow mineral supplements used on 24 Irish dairy farms

| Item1 |  | Median | Range | CV2 (%) | n3 |
| --- | --- | --- | --- | --- | --- |
| Multi-mineral Powder | 21(16) |
| Ca |  | 1.5 | 0.7-12.8 | 126.9 | 16(11) |
| Mg |  | 28.0 | 3.0-50.0 | 34.3 | 21(16) |
| Na |  | 9.0 | 2.4-18.0 | 40.9 | 20(16) |
| P |  | 3.5 | 2.0-14.0 | 60.5 | 20(13) |
| S |  | 1.2 | - | - | 1 (1) |
| Co |  | 100.0 | 75.0-100.0 | 6.1 | 20(13) |
| Cu |  | 3000.0 | 2640.0-3500.0 | 5.7 | 20(13) |
| I |  | 500.0 | 400.0-500.0 | 4.7 | 20(13) |
| Mn |  | 3000.0 | 1333.0-3350.0 | 19.9 | 20(13) |
| Se |  | 50.0 | 27.0-50.0 | 11.5 | 20(13) |
| Zn |  | 4500.0 | 2667.0-8050.0 | 30.8 | 20(13) |
| Magnesium Sources | 4(2) |
| MgCl2 |  | 47.0 | 47.0-47.0 | 0.0 | 3(2) |
| Mineral Licks | 2(2) |
| Ca |  | 4.0 | - | - | 1(1) |
| Mg |  | 6.8 | 6.5-7.0 | 5.2 | 2(2) |
| Na |  | 11.0 | 4.0-18.0 | 90.0 | 2(2) |
| P |  | 3.3 | 1.0-5.5 | 97.9 | 2(2) |
| Co |  | 60.0 | 20.0-100.0 | 94.3 | 2(2) |
| Cu |  | 2000.0 | 1000.0-3000.0 | 70.7 | 2(2) |
| I |  | 350.0 | 200.0-500.0 | 60.6 | 2(2) |
| Mn |  | 1500.0 | 1000.0-2000.0 | 47.1 | 2(2) |
| Se |  | 35.0 | 20.0-50.0 | 60.6 | 2(2) |
| Zn |  | 3000.0 | 3000.0-3000.0 | 0.0 | 2(2) |
| Water Source | 2(2) |
| Mg |  | 3.45 | 0.5-6.4 | 98.7 | 2(2) |
| Na |  | 0.6 | - | - | 2(1) |
| P |  | 0.2 | - | - | 2(1) |
| Co |  | 270.0 | - | - | 2(1) |
| Cu |  | 12525.0 | - | - | 2(1) |
| I |  | 266.0 | - | - | 2(1) |
| Mn |  | 6000.0 | - | - | 2(1) |
| Se |  | 264.0 | - | - | 2(1) |
| Zn |  | 20750.0 | - | - | 2(1) |
| Salt | 1(1) |
| Na |  | 38.0 | - | - | 1(1) |
| I |  | 1000.0 | - | - | 1(1) |
| Zn |  | 2000.0 | - | - | 1(1) |

1Macrominerals (Ca, Mg, Na, P) in % of DM, trace minerals (Co, Cu, I, Mn, Se, Zn) in mg/kg of DM.

2CV = Coefficient of variation.

3Number of farms that incorporated each item in their mineral supplement regime and number of different products in parentheses.

References

1. National Academies of Sciences, Engineering, and Medicine; Division on Earth and Life Studies; Board on Agriculture and Natural Resources; Committee on Nutrient Requirements of Dairy Cattle. Nutrient requirements of dairy cattle: eighth revised edition. 2021.

2. Irish Cattle Breeding Federation: New Maintenace Sub index In Brief. https://www.icbf.com/new-maintenace-sub-index-in-brief/ (2020). Accessed 5 Mar 2024.  

3. Ramsbottom G. Maintenance Excel Calculator. Available: https://www.icbf.com/new-maintenace-sub-index-in-brief/ (2020). Accessed 5 Mar 2024.
